# Electrical impedance as an indicator of microalgal cell health

**DOI:** 10.1038/s41598-020-57541-6

**Published:** 2020-01-27

**Authors:** Jianye Sui, Fatima Foflonker, Debashish Bhattacharya, Mehdi Javanmard

**Affiliations:** 1 Department of Electrical and Computer Engineering, Rutgers University, Piscataway, NJ 08854 USA; 2 Department of Biochemistry and Microbiology, Rutgers University, New Brunswick, NJ 08901 USA

**Keywords:** Cytological techniques, Functional genomics, Bionanoelectronics, Biosensors, Cell biology

## Abstract

Separating specific cell phenotypes from a heterotypic mixture is a critical step in many research projects. Traditional methods usually require a large sample volume and a complex preparation process that may alter cell property during the sorting process. Here we present the use of electrical impedance as an indicator of cell health and for identifying specific microalgal phenotypes. We developed a microfluidic platform for measuring electrical impedance at different frequencies using the bacterium-sized green alga *Picochlorum* SE3. The cells were cultured under different salinity conditions and sampled at four different time points. Our results demonstrate the utility of electrical impedance as an indicator of cell phenotype by providing results that are consistent with known changes in cell size and physiology. Outliers in the cell data distribution are particularly useful because they represent phenotypes that have the ability to maintain size and/or membrane ionic permeability under prolonged salt stress. This suggests that our device can be used to identify and sort desired (e.g., experimentally evolved, mutant) cell phenotypes based on their electrical impedance properties.

## Introduction

An important branch of functional genomics relies on the bioinformatic analysis of bulk transcriptomic data (e.g., RNA-seq reads from Illumina or Iso-Seq data from PacBio platforms) to identify pathways involved in processes such as the cell cycle, stress and disease response, and development^1–3^. Although well understood and relatively easy to apply, these methods are nonetheless expensive and provide average gene expression data based on the analysis of millions of cells per sample. To achieve single-cell resolution of gene expression patterns requires the more specialized tools of single cell transcriptomics that may be limited to smaller sample sizes due to the costs of generating individual libraries for 100 s or 1000 s of cells, followed by high-throughput sequencing^4,5^. Given these considerations, there is a need to develop tools with single cell resolution that provide meaningful insights into cell health and can be applied to millions of cells at low cost. If such an approach does not require existing reference genome data (preferable for RNA-seq approaches), then it can be applied to a variety of non-model systems (algal or microbial) that are of high importance in natural settings. Such a tool should be portable and its use not limited to laboratories or highly trained specialists. Here we describe a microfluidic platform for measuring cell health at the single cell level that addresses many of the shortcomings of conventional approaches.

Our approach builds on the growing interest in electrical analysis of biological cells. Particularly attractive is the ease of operation, rapid processing time, non-necessity of labeling, and the potential of miniaturization of these methods. For these reasons, electrical properties of cells have been investigated and utilized in a broad array of fields such as disease diagnosis^6–9^, environmental monitoring^10,11^, food safety^12^, and in applications such as cell identification and separation^13–15^. Specifically, electrical impedance spectroscopy has been used to analyze cell electrical properties^16–20^. Impedance measurement is based on the changes in conductivity and permittivity in a medium due to the presence of cells. Electrical impedance measured at different frequencies provides different types of information about cells, including cell size and membrane and cytoplasm electrical properties^21^. Impedance indicates cell size at lower frequencies around several hundred kilohertz, whereas it can be used to interpret membrane reactance and cytoplasm conductance at higher frequencies^21,22^. With the aid of microfluidic flow cytometry technology, impedance spectroscopy requires a smaller sample volume when compared to traditional methods, while maintaining high sensitivity^23–25^. Moreover, it simplifies the preparation process that may alter cell properties during the sorting step. Morgan’s group used microfluidic impedance cytometry to discriminate T-lymphocytes, monocytes, and neutrophils in blood with high accuracy^26^. Based on the electrical impedance, cells with particular properties can be sorted for downstream analysis. Haandbæk and co-authors reported a cytometer with the capability of wide frequency range measurement and characterized two different types of yeast cells based on dielectric properties at four frequencies^27^. There are some known connections between electrical properties of cells and their biological status, such as viability. Song *et al*. differentiated live and dead *Dunaliella salina* cells with a capacitive microfluidic sensor^28^. In spite of these promising results, the use of electrical impedance for cell health screening is poorly developed.

Here, we present a novel method to study algal cell phenotype using electrical impedance cytometry at multiple frequencies, providing an instantaneous snapshot of organism dielectric properties at the single cell level. We investigated the frequency-dependent impedance of bacterium-size (i.e., 2–3 µm cell diameter) green algal cells (*Picochlorum* SE3, Chlorophyta)^29,30^. The algae were cultured in three different salinity conditions and sampled at four different time points over a wide frequency range using a multi-frequency lock-in amplifier that was utilized in conjunction with a microfluidic channel. We demonstrate the utility of electrical impedance as a phenotype indicator that reflects the change in size and permeability of cells under different salt stresses.

## Results

### Microfluidic sensor design and electrical impedance analysis

**Figure 1:**
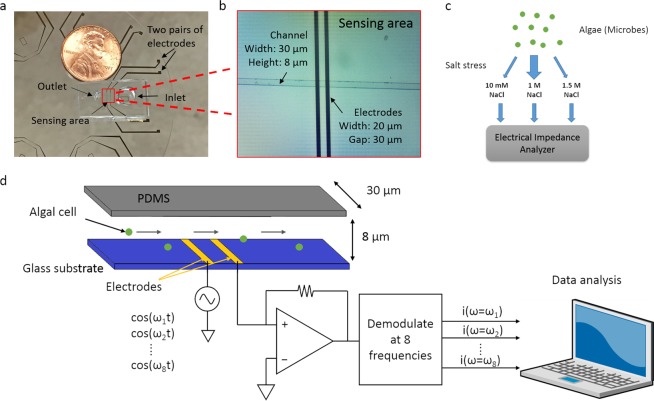
Overview of strategy. (**a**) Image of the device whereby a soft-lithography made PDMS microfluidic channel is bound onto a glass wafer patterned with two pairs of sensing electrodes. (**b**) Microscope image of the channel and electrodes. (**c**) Diagram showing the experimental design of the cell impedance experiments in which *Picochlorum* SE3 cells were cultured under widely different salinity conditions (10 mM, 1.5 M NaCl) after being acclimated to 1 M NaCl, and sampled at 4 different time points (1 h, 5 h, 1 d, and 5 d). After culturing, all cells were washed three times in PBS buffer and injected into the electrical impedance analyzer to collect the data. (**d**) Schematic diagram of the electrical impedance measurement. Algal cells were introduced into the channel from the inlet well. When cells flowed through the sensing region, they blocked part of the ions conducting current between the two electrodes. As a result, the impedance changed in this region. This change was captured by a lock-in amplifier at eight different frequencies. The data were transferred to the attached computer for downstream analysis.

**Figure 2:**
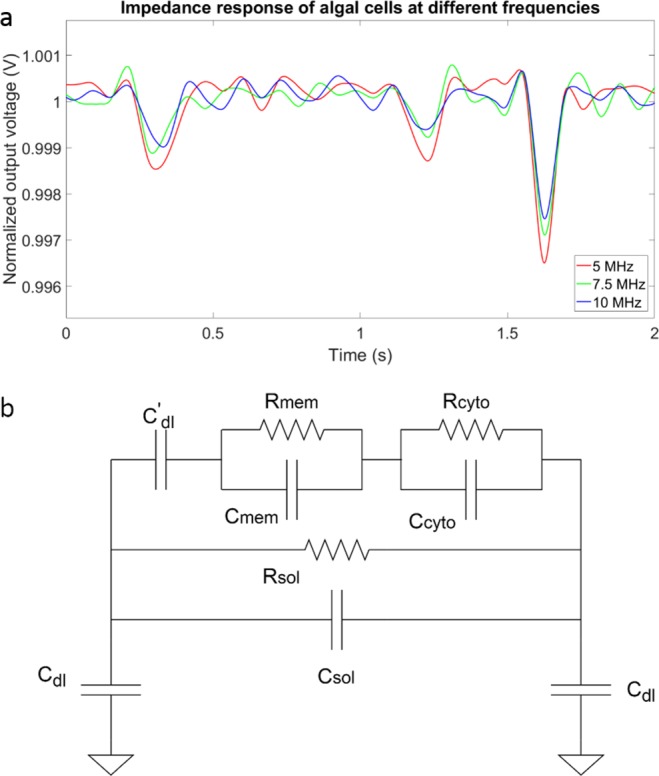
Impedance response analysis. (**a**) Representative data for algal cells flowing through the sensing electrodes, measured at 5 MHz, 7.5 MHz and 10 MHz. The line colors denote the different frequencies used (see legend) and the three peaks denote three cells flowing through the sensing area in this 2-second window. (**b**) Impedance model of the cytometer system with the algal cell present. C’dl is the double layer capacitance of the cell. The impedance of cell is in parallel with the solution resistance and capacitance. Cdl is the double layer capacitance of the electrodes.

We built a microfluidic sensor to perform multi-frequency impedance cytometry to capture the impedance information of algal cells. As shown in Fig. 1, the instrument comprises two components, two pairs of coplanar golden electrodes deposited on a glass substrate and a polydimethylsiloxane (PDMS) microfluidic channel. To enhance sensitivity and prevent blockage, the channel dimension was 30 μm in width and 8 μm in height. The width of the two electrodes was 20 μm and the gap between them was 30 μm. In the experiments we describe below, only one pair of electrodes was used for measurement. When a cell flows through the sensing region, it occludes a portion of the ionic current conducting between the two electrodes. Thus, the current decreases, and conversely the impedance increases. The closer the dimensions of the sensing region to the size of algal cells, the more current is obstructed and the larger the impedance change. However, blockage is more likely to happen when the channel size is reduced. A commercial multi-frequency lock-in amplifier (Zurich Instruments HF2A, Zurich, Switzerland) was used to capture the impedance change simultaneously at eight different frequencies (ranging from 500 kHz to 30 MHz). Output voltage is proportional to impedance between the two electrodes (sensing region). As described above, when a cell flows through the sensing region, the current between two electrodes decreases, thus the output voltage of the lock-in amplifier decreases and a negative peak is observed. The larger the output voltage peak amplitude, the greater the cell impedance. The peak amplitude is calculated as the difference between the output voltage baseline and the minimum value of the peak. The typical impedance change (output voltage) at different frequencies (5 MHz, 7.5 MHz and 10 MHz) when a cell passes by in a 2-second time window is shown in Fig. 2a. Traces were normalized using the baseline to allow between-frequency comparison. Previous work from Sun *et al*.^18^ and Gawad *et al*.^22^ demonstrated that a cell can be modeled as a membrane resistance, in parallel with membrane capacitance, and then in series with the cytoplasm resistance, in parallel with cytoplasm capacitance (Fig. 2b). The C_dl_ and C’_dl_ are the double layer capacitance that surrounds the electrodes and algal cells, respectively, and they will have less impact on impedance when the frequency exceeds several kilohertz. As the frequency increases above 5 MHz, the membrane capacitance gradually gets shorted and thereby creates a path for conducting the current. As a result, the cell becomes more permeable to the electric field generated by the electrodes, and has lower impedance. As the frequency increases, cell impedance depends more on properties of the cytoplasm. In contrast, under a low frequency range (<500 kHz), cell size dominates the impedance^21^. To minimize the effect of variation in cell size on impedance, we defined cell transparency as the ratio of voltage peak intensity (voltage peak amplitude) at higher frequency over peak intensity at 500 kHz, whereby the peak intensity corresponds to size. The cell transparency reflects cell properties independent of size and also denotes the extent of similarity between the cell and the background solution in the electric field.

### Impedance analysis of algal cell viability

**Figure 3:**
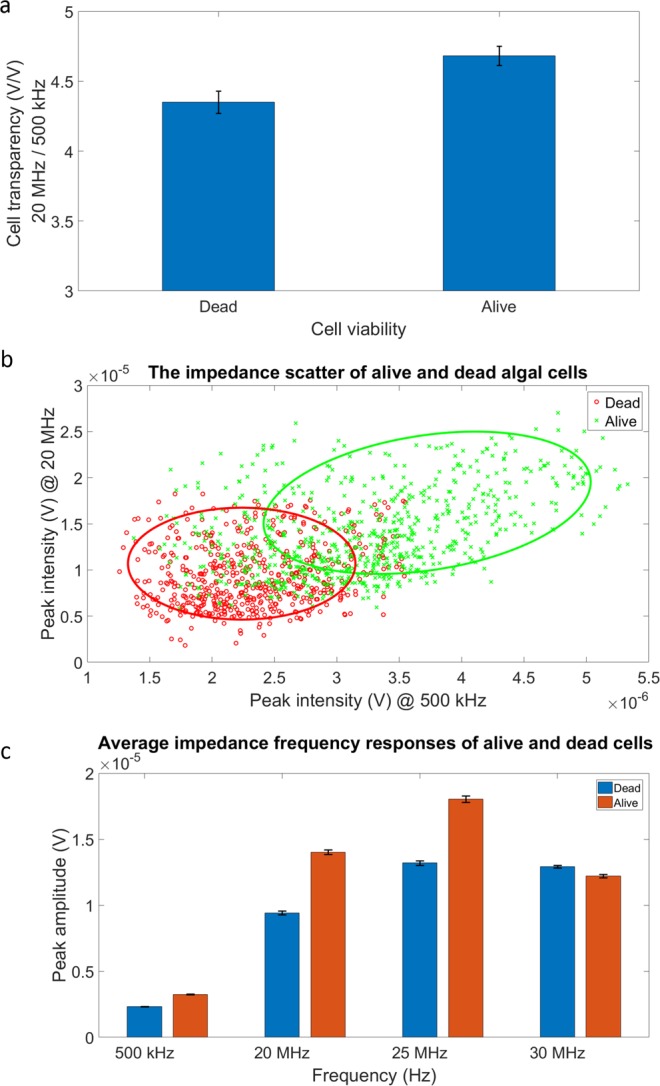
Impedance response of live and dead algal cells. (**a**) Average cell transparency calculated using peak intensity measured at 20 MHz over peak intensity measured at 500 kHz. (**b**) The impedance scatter of live and dead algal cells. The x-axis shows the peak intensity at 500 kHz, which indicates cell size. The y-axis shows the peak intensity at 20 MHz, which indicates algal cytoplasmic permeability. (**c**) Average impedance frequency response of live and dead algal cells. The impedance responses were measured at 500 kHz, 20 MHz, 25 MHz, and 30 MHz.

Initially, we studied the impedance responses of live and dead *Picochlorum* SE3 cells. The algae were killed by treating them with heat for 1 h. Multi-frequency impedance flow cytometry was performed to measure the impedance of cells. Both live and dead cells were diluted in 50 μL of 1X phosphate buffered saline (PBS) immediately before the measurement to allow higher sensitivity. The results of these analyses are shown in Fig. 3. As apparent in Fig. 3a, the mean cell transparency of live cells was greater than that of dead cells. The transparency was calculated using average peak intensity measured at 20 MHz over average peak intensity measured at 500 kHz. The peak intensity at 20 MHz reflected more the cytoplasmic permeability, whereas the peak intensity at 500 kHz was affected primarily by cell diameter^21^. These results indicate that dead cells are more transparent to the surrounding electric field, because ions in the media can flow more freely through the membrane. The impedance scatter plot of live and dead cells is shown in Fig. 3b. Dead cells exhibit smaller peak intensity at 500 kHz, implying that they are smaller in size compared to live cells. The cytoplasmic permeability of dead cells was greater than that of live cells, as can be concluded from the smaller peak intensity of dead cells at 20 MHz. In addition, dead cells have a narrower distribution compared with live cells. In other words, dead cells exhibit more homogeneity in terms of size and permeability. With regard to the frequency response of live and dead cells (Fig. 3c), the peak intensity difference was small at 500 kHz but became larger as the frequency increased to 25 MHz. This difference became small again at 30 MHz. This pattern illustrates that the size difference between live and dead cells is smaller compared to the difference in cytoplasmic permeability, as depicted in Fig. 3b. These results demonstrate the utility of electrical phenotype for studying not only the viability of algal cells, but also the health of a population.

### Algal cell stress analysis using impedance flow cytometry

#### Algal cells stressed at different salinities

**Figure 4:**
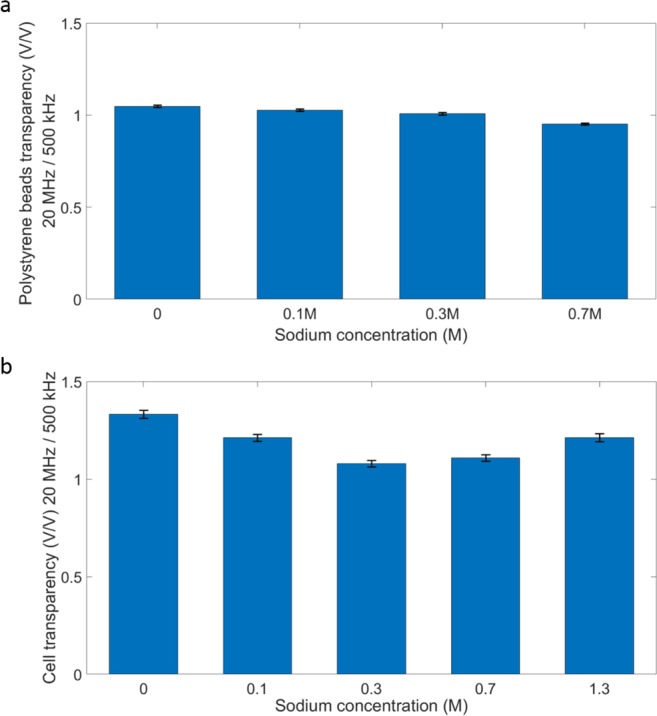
Impact of different culture salt conditions on polystyrene bead (PS) and algal cell impedance. Algal cells were cultured in 0 M, 0.1 M, 0.3 M, 0.7 M, and 1.3 M NaCl amended medium for 1 h. PS beads were used as control and immersed in PBS medium amended with 0 M, 0.1 M, 0.3 M, and 0.7 M NaCl. Both beads and cells were washed and resuspended in PBS before the impedance measurements were made. (**a**) Average PS bead transparency calculated using peak intensity measured at 20 MHz over peak intensity measured at 500 kHz. (**b**) Average cell transparency calculated using peak intensity measured at 20 MHz over peak intensity measured at 500 kHz. These results show that the PS bead transparency changed very little in the different salt treatments, whereas algal cell transparency decreased significantly from 0 M to 0.3 M and then increased from 0.3 M to 1.3 M.

We measured the impedance responses of *Picochlorum* SE3 cells cultured in media of different salinities (0 M, 0.1 M 0.3 M, 0.7 M, and 1.3 M NaCl) for 1 h using multi-frequency impedance flow cytometry. For this approach, 3 mL of algal cells were centrifuged, washed three times with 1X PBS and diluted in 50 μL PBS. For the control, we measured the impedance response of polystyrene (PS) beads that were incubated in PBS amended with different amounts of salt (0 M, 0.1 M 0.3 M, and 0.7 M NaCl) for one hour. The beads were handled in the same way as the algal cells with regard to centrifugation, the wash and dilution process prior to the experiments. Figure 4 shows impedance measurements of average cell transparency using PS beads and of algae incubated in media of different salinities. The transparency was calculated using peak intensity measured at 20 MHz over peak intensity measured at 500 kHz. The mean transparency of PS beads was similar across treatments, whereas the mean transparency values of algal cells under different salt conditions were highly differentiated. Cell transparency decreased as the salinity increased from 0 M to 0.3 M and then increased from 0.3 M to 1.3 M. The moderate salinity level did not impact the property of PS beads and hence bead transparency was unaltered. In contrast, algal cells need to regulate the flow of sodium ions in the different salinity environments to maintain ionic homeostasis^31^. As a result, in addition to changes in cell size, the algal cell membrane and cytoplasm were altered to accommodate the changing environment^32^. This series of responses changed several frequency-dependent electrical properties of the membrane and the cytoplasm, such as surface conductance, dielectric permittivity, and the charging profile, which had an impact on electrical impedance and cell transparency, that changed accordingly^33^. At a salinity of around 0.3 M NaCl, the algae were apparently able to maintain ionic homeostasis more effectively. Therefore, the membrane ionic permeability was high and resulted in low cell transparency. By comparing the results of PS and algal cells, we validated that our sensor had the ability to detect different levels of algal salt stress using electrical impedance.

#### Algal cells stressed for different times

**Figure 5:**
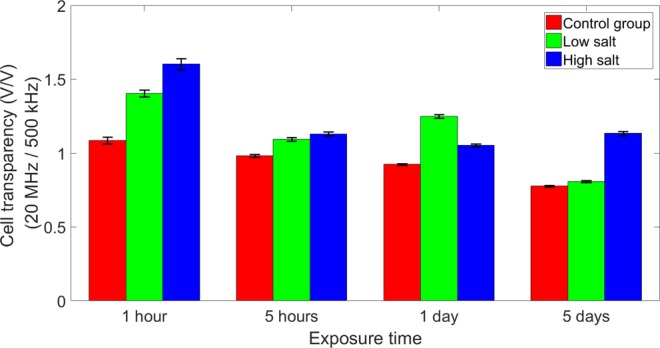
Impact of time on algal cell impedance. The plot shows the average cell transparency calculated using peak intensity measured at 20 MHz over peak intensity measured at 500 kHz.

We investigated if culturing algal cells in different salinity conditions for different periods of time had an impact on the impedance response. Algal cells were cultured in three different salinity conditions (0.01 M, control: 1 M, and 1.5 M NaCl) and sampled at four different time points (1 h, 5 h, 1 d, and 5 d). Cells cultured in 1 M salinity were used as the control^31^. All samples were washed 3X to remove the residual original medium. We measured the impedance of algal cells using impedance flow cytometry. The cell transparency was calculated as the ratio of peak amplitude at 20 MHz over peak amplitude at 500 kHz. As shown in the average cell transparency (Fig. 5), cell impedance response in terms of transparency was different at 1 h. The high and low salt groups were significantly less transparent than the control group. Cells in these two groups presumably were more highly stressed in the short-term and the membrane became less permeable with respect to ion transport. After 5 hours, and up to 1 day, cells adapted better to the environment and thus membrane permeability again normalized. Hence, cells were more transparent to the surrounding environment and the electric field generated by the electrodes. After 5 days, the high salt group could not recover from the long-term salinity stress and growth of these cells was greatly diminished^31^. Therefore, the cytoplasm of this group was less conductive and cells become less transparent when compared to the other two groups.

**Figure 6:**
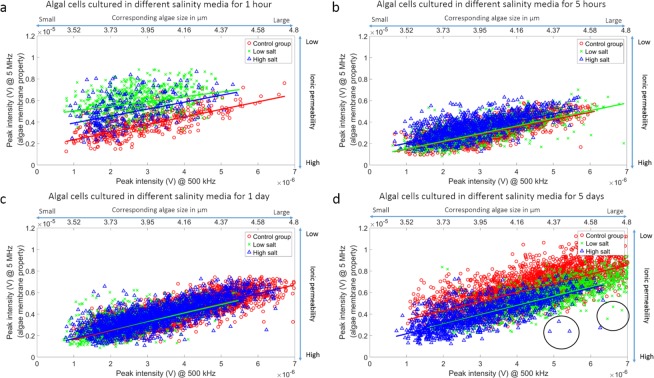
The impedance scatter of individual algal cells cultured in different salinity media for 1 h (**a**), 5 h (**b**), 1 d (**c**), and 5 d (**d**). The x-axis shows the peak intensity at 500 kHz, which indicates cell size. The y-axis shows the peak intensity at 5 MHz, which indicates algal membrane properties, in particular, ionic permeability. The solid line shows the average cell transparency in terms of size. After 1 h, cell transparency of the low salt and high salt groups were above that of control. The cell transparency of all three groups became similar to each other. However, after 5 d, cells under both test conditions were less well adapted and showed a similar, relatively high membrane permeability with low salt conditions cells having a larger cell size. The cells marked with the black circles had relatively high membrane permeability and were larger in size under either low or high salt conditions after 5 d exposure.

Figure 6 shows the impedance scatter plot of individual algal cells cultured in different salinity media for 1 h, 5 h, 1 d, and 5 d. The solid line is the average cell transparency in terms of size. The impedance peak intensity of algal cell at 500 kHz illustrates cell size. The second x-axis on the top of the images shows the corresponding cell size, which was determined through light microscopy. The peak intensity at 5 MHz indicates ionic permeability of the cell membrane^21^. At 1 h, cells under low or high salt stress alter their membrane to be less permeable than cells in the control group while maintaining a similar size to withstand the shock. After 5 h and 1 d, all cells in different media had similar properties, except the low salt group, which had a slightly larger size. However, on day 5 cells under both high salt and low salt conditions were less well adapted and showed a similar, relatively high permeability with low salt conditions cells having a larger cell size. The membrane of cells in both conditions was more permeable than that of cells in the control medium. Outliers in the data scatter plot were of interest because they represent phenotypes such as algae that have the ability to maintain cell size and/or membrane ionic permeability under prolonged salt stress. For example, the outlier cells marked with black circles maintain relatively high permeability and large cell size under both low and high salt conditions after the 5-day exposure. With the addition of an integrated sorter, our device potentially could be used to isolate cells with specific impedance properties for downstream analysis.

## Discussion

*Picochlorum* SE3 is a remarkably versatile coccoid green alga that can tolerate a wide salinity range^34^. Strategies for maintaining ionic homeostasis are critical for the survival of *Picochlorum* SE3 in its natural habitat of a brackish water coastal lagoon that is subject to large fluctuations in salinity through evaporation, precipitation, and tidal influx of seawater. The San Elijo Lagoon system in California where this strain was isolated has salinities that range from 108.3‰ in the dry season to freshwater levels (1.7‰) in the rainy winter season. Nutrients such as phosphate, nitrate, nitrite, and ammonium also show extreme variation. *Picochlorum* SE3 is present year-round in this fluctuating environment and therefore has evolved mechanisms to deal with this long-term stress. For example, this alga encodes six copies of the AtNHX8/salt overly sensitive 1 (SOS1) gene (compared to one in the marine green alga *Ostreococcus tauri*) and contains several horizontally transferred bacterial genes that play roles in abiotic stress responses^30,31^. The overall “genetic toolkit” (e.g., transcriptomic response) that *Picocholrum* SE3 deploys to survive salt stress is however shared by many other algae and plants and is well understood^35–37^.

Nonetheless, without analyzing impedance phenotypes associated with different gene knockouts, we have no direct way to connect cell impedance with genetic or transcriptomic data. Therefore, the inferences we make about how electrical impedance intersects with algal biology are speculative but broadly consistent with existing physiological and RNA-seq results from *Picochlorum* SE3^3,30,31^. Specifically, the growth rate of this alga, when pre-conditioned to 1 M NaCl peaks at 100 mM NaCl and is reduced drastically as the culture salinity is increased above 1 M NaCl. Analysis of the cultures shows that the PSII quantum efficiency (proportional to *Fv*/*Fm*) is lessened in the short-term (2 h) at both lower and higher salt concentrations. The correlation of *Fv*/*Fm* and the growth rate, based on salt conditions after 24 h suggests that energy resources normally devoted to growth are instead used to maintain osmotic balance, with both low and high salinity having an impact^31^. Higher observed growth rates under low salt indicates that these conditions favor enhanced growth after an initial acclimation period, whereas high salt stress does not allow recovery of the growth rate over the longer-term (up to several days; for more details, see^31^). Finally, an intriguing finding in our study is the strict control of cell size apparent in control populations as well as in the different salt treatments that ranges from 4.5–4.8 μm (Fig. 6). Even after 5 d when the low and high salt populations have differentiated in terms of cell size, they remain within the bounds formed by the control group (Fig. 6d). These results suggest that cell size is “hard-wired” in *Picochlorum* SE3 and that cells explore (and not escape) the range of possible sizes under different salt conditions.

In this work, we presented the use of electrical impedance as an indicator of cell health and for identifying specific microalgal phenotypes by implementing multi-frequency impedance flow cytometry. Multi-frequency impedance responses provide information about the size and membrane and cytoplasm ionic permeability of algal cells. These properties will change as cells undergo salt stress for different time periods. Large cell size exhibits a large impedance response at lower frequency (~500 kHz)^21^. High ionic permeability produces a small impedance response at higher frequency (>5 MHz). By investigating the impedance at different frequencies, we were able to resolve differences in cell size, infer membrane and cytoplasm ionic permeability, identify outliers in the cell population distribution, and estimate the overall level of cell stress. Our method provides a novel high-throughput approach to study algal (and potentially, any microbial) cell health. In addition, this approach can be used to identify and sort desired (e.g., experimentally evolved, mutant) cell phenotypes based on their electrical impedance.

Several optimization steps could be made to the device to further improve the accuracy of this method for identifying and sorting cell phenotypes. Implementing multi-electrodes for differential configuration can remove some of the interference and noise from the environment during measurement, such as the baseline shift and white noise, and thus can enhance the signal to noise ratio. Incorporating temperature sensors in the microfluidic channel can correct day-to-day variation due to temperature differences that can affect conductivity of the medium and hence cell impedance. These advances could also reduce the number of algal cells used for analysis and the testing time. In summary, our method captures impedance changes in the microfluidic channel and relies on a portable readout instrument, which can also be integrated on a chip to further minimize the size in future iterations. Impedance flow cytometry enables measuring hundreds of cells in minutes to save analysis time. Moreover, it requires small sample volumes and no complex sample preparation process. Given these advantages, our method provides an opportunity for the rapid *in situ* analysis of cell phenotype, and in the future, sorting of cells with desired properties.

## Methods

### Algal cell preparation

*Picochlorum* SE3 was cultivated as previously described in artificial seawater^30^ based Guillard’s f/2 medium^38^ without silica (f/2 ASW–Si). The cells were grown at 25 °C under continuous light (100 μE m^−2^ s^−1^) on a rotary shaker at 100 rpm (Innova 43, New Brunswick Eppendorf).

### Device fabrication and integration

The device consists of two pairs of gold electrodes on a glass substrate and a polydimethylsiloxane (PDMS) microfluidic channel fabricated using microfabrication technology. Electrodes were patterned on a 3-inch glass wafer using standard photolithography procedures. The process started with wafer cleaning using acetone, methanol and DI water. Thereafter, a thin layer of positive photoresist (AZ5214, MicroChemicals GmbH) was spin coated on the wafer. After pre-bake, mask and wafer alignment, UV exposure, development and post-bake, the desired pattern was transferred from the mask to the wafer. The metals, 5 nm chromium and 100 nm gold, were deposited sequentially using electron beam evaporation, whereby the chromium layer was used for enhancing the adhesion of gold film on glass. Unintended parts were lifted off by submerging the wafer in acetone. The resultant electrodes were 20 μm in width and the gap between two electrodes was 30 μm. The SU-8 (negative photoresist) silicon mold for microfluidic channel was fabricated using standard soft lithography procedures as described in the literature^39–41^. The size of the channel is 30 μm in width and 8 μm in height. The channel pattern was transferred from the mold to a PDMS slab using the following process. Mixed 10:1 PDMS polymer and curing agent (Sylgard 184, Dow Corning) sufficiently. Then, poured the mixture onto the channel mold, degassed to remove bubble in the mixture and baked at 80 °C for 30 minutes to allow for curing. Afterwards, the PDMS channel was peeled off and two holes were punched through the PDMS to be used as inlet reservoir (5 mm in diameter) and outlet reservoir (1.2 mm in diameter). The microfluidic channel and the electrodes on glass substrate were covalently bonded by treating the two surfaces with oxygen plasma.

### Multi-frequency impedance flow cytometry

Multi-frequency impedance flow cytometry was done to capture the impedance response of algal cells at 8 different frequencies, ranging from 500 kHz to 30 MHz. The algal cells were initially cultured in Guillard’s f/2 medium emended with different NaCl amounts (0 M, 0.01 M, 0.1 M, 0.3 M, 0.7 M, 1 M, 1.3 M, and 1.5 M) for different time periods (1 hour, 5 hours, 1 day, and 5 days) and then analyzed. Cells in 3 mL of the original culture were sampled, spun down and the culture buffer was replaced with 1x phosphate buffered saline (PBS). Samples were washed in PBS 3 times to remove the original culture buffer. Finally, samples were diluted in 50 μL PBS, which provided a continuous flow in the cytometry experiment for more than 15 min without using a noisy syringe pump. The impedance flow cytometry started with making the microfluidic channel hydrophilic by implementing an oxygen plasma treatment. The PBS was injected into the channel to preserve the hydrophilicity until the measurement started. Fluid was driven by capillary force, and the pressure gradient induced by the fluid height difference between inlet and outlet. PBS was withdrawn from the channel and algal cells were introduced from the inlet. The impedance across two electrodes was changed when a cell flowed through the sensing region, because the electrical field was blocked by the cell. The impedance changes at 8 different frequencies were captured by a commercial lock-in amplifier (Zurich Instruments HF2A, Zurich, Switzerland). A superposition of 8 different frequency AC signals generated by the same lock-in amplifier was used to excite the electrodes. The data were demodulated, then sent to a local computer and analyzed using MATLAB (MathWorks, Natick, MA, USA). To minimize the impact of noise and interference from the environment on impedance measurement, the device was placed in a metal box during the experiment.

## Data Availability

The datasets generated during and/or analysed during the current study are available from the corresponding author on reasonable request.
